# Erythropoietin Resistance Development in Hemodialysis Patients: The Role of Oxidative Stress

**DOI:** 10.1155/2022/9598211

**Published:** 2022-04-14

**Authors:** Jovana Joksimovic Jovic, Svetlana Antic, Tomislav Nikolic, Kristina Andric, Dejan Petrovic, Sergey Bolevich, Vladimir Jakovljevic

**Affiliations:** ^1^University of Kragujevac, Faculty of Medical Sciences, Department of Physiology, Svetozara Markovica 69, 34000 Kragujevac, Serbia; ^2^Military Medical Academy, Clinic for Nephrology, Crnotravska 17, 11000 Belgrade, Serbia; ^3^University of Kragujevac, Faculty of Medical Sciences, Department of Internal Medicine, Svetozara Markovica 69, 34000 Kragujevac, Serbia; ^4^Clinical Center Kragujevac, Clinic for Urology and Nephrology, Center for Nephrology and Dialysis, Zmaj Jovina 30, 34000 Kragujevac, Serbia; ^5^University of Kragujevac, Faculty of Medical Sciences, Department of Dermatovenerology, Svetozara Markovica 69, 34000 Kragujevac, Serbia; ^6^Clinical Center Kragujevac, Center for Dermatology, Zmaj Jovina 30, 34000 Kragujevac, Serbia; ^7^Department of Human Pathology, 1st Moscow State Medical University IM Sechenov, Moscow, Russia

## Abstract

Oxidative stress (OS) is considered a significant risk factor for the development of anemia in patients treated by regular hemodialysis (HD). Moreover, OS represents a risk factor for the development of erythropoietin (EPO) resistance in these patients. The aim of this study was to examine the role of OS regarding EPO resistance development in patients treated by regular HD. 96 patients treated with standard HD and on-line hemodiafiltration were included in this study. The patients were treated with short-acting and long-acting EPOs for anemia. The concentration of superoxide anion radical, hydrogen peroxide, thiobarbituric acid reactive substances, and nitric oxide in the form of nitrites and the activity of catalase, superoxide dismutase and reduced glutathione were measured in patients' blood spectrophotometrically. Standard biochemical analysis, inflammatory markers, nutritional status, HD parameters, and erythropoietin resistance index were also determined. Patients with resistance to short-acting EPO had significantly lower concentration of hemoglobin in the blood and hematocrit value, a significantly higher serum ferritin concentration, and significantly lower catalase activity in erythrocytes than patients without EPO resistance. Patients with resistance to long-acting EPO have a significantly lower hemoglobin concentration in the blood, hematocrit values, and serum concentration of prealbumin and vitamin D, as well as significantly higher concentration of C-reactive protein, superoxide anion, and hydrogen peroxide concentration than those without resistance. OS significantly contributes to EPO resistance development. OS, higher ferritin and CRP levels, lower hemoglobin, hematocrit and prealbumin levels, and vitamin D deficiency represent significant risk factors for EPO resistance development in HD patients.

## 1. Introduction

Cardiovascular diseases (CVDs) remain the leading cause of death in patients undergoing regular hemodialysis (HD). Beside traditional risk factors for CVDs development in HD patients , there are numerous nontraditional risk factors, such as oxidative stress (OS), microinflammation, malnutrition, endothelial dysfunction, effects of uremic toxins, hyperhomocysteinemia, anemia, hypervolemia, vitamin D deficiency, as well as secondary hyperparathyroidism. Regarding end-stage renal disease (ESRD), parallel with kidney dysfunction, renal anemia primary manifests due to impaired erythropoietin (EPO) production by specialized peritubular cells [1]. However, in HD patients, anemia was related to other entities, such as hemorrhages, impaired erythropoiesis, or oxidative damage of erythrocytes [2]. The main cause of anemia in patients undergoing regular HD was EPO deficiency, and the main clinical consequences were described as development of CVDs, decreased quality of life, and increased cardiovascular mortality risk. Despite the administration of EPO (original formulations, EPO biosimilars) at appropriate doses, about 5-10% of patients treated by regular HD will develop EPO resistance [3–5].

Maintaining the balanced redox status in organism is crucial for homeostasis in healthy subjects as well as in HD patients. Various factors could influence generation of reactive oxygen species (ROS) in HD patients, including malnutrition, microinflammation, vitamin D deficiency, and iron status. These factors could significantly contribute to poor prognosis in ESRD patients treated by HD. The literature data showed that OS could be marked as a significant risk factor for the development of anemia in patients treated by regular HD due to EPO resistance [2]. Also, it was known that OS could be diminished by improving anemia in HD patients [6]. However, in these patients, anemia was commonly treated by EPO stimulating agents (ESA) or by iron therapy, which could individually affect oxidative status in patients [7]. Nevertheless, the clear relationship between OS and anemia in HD patients was not completely revealed yet. The aim of the study was to assess the role of OS in EPO resistance in patients treated with long- and short-acting EPO and to analyze the risk factors for EPO resistance development in patients treated by regular HD.

## 2. Material and methods

### 2.1. Ethical Approval

The study was conducted in accordance to the ethical principles of the Declaration of Helsinki for medical research. This study was approved by the Ethics Committee of the Clinical Center Kragujevac (number: 01/19/107, date: 23.01.2019.) and all subjects gave their written informed consent to participate.

### 2.2. Patients and Study Design

The study included 96 patients of both sexes, treated with regular HD and online hemodiafiltration at the Center for Nephrology and Dialysis of the Clinical Center Kragujevac, Kragujevac, Serbia. The patients were treated by regular HD (85 patients) or online hemodiafiltration (11 patients) three times a week for 4 hours (12 h per week), for a period longer than three months. The procedures were performed using the “high-flux” membranes (for regular HD: polysulfone high-flow membrane, surface area 1.4-1.8 m^2^; for hemodiafiltration: polysulphone “high-flux” membrane, surface area 2.0-2.4 m^2^), on machines with controlled ultrafiltration type Fresenius 5008S, Gambro Artis, and B Braun, with an average blood flow volume of Qb =219.06 ± 26.44 mL/min and average volume dialysate flow rate of Qd =500.00 mL/min. A standard ultra-pure HD solution (endotoxin concentration, E <0.03 EU/mL) was used, with a calcium concentration of 1.75 mmol/L (PGS21), 1.50 mmol/L (PGS25), and 1.25 mmol/L (PGS27). Convective volume (Vconv) in patients treated with hemodiafiltration online was 17 L per session. Unfractionated heparin was used for anticoagulation of the extracellular circulation. The average monthly dose of unfractionated heparin for single HD was 4385.42 ± 591.52 IU. The patients with proven active hemorrhage, active systemic inflammation or infection (mean leukocyte count was 6.98 ± 1.74 × 10^9^/L), uncontrolled malignancies, or treated by immunosuppressive and antioxidant medications were excluded from the study.

All patients were treated with ESA, and depending on the type, the patients were divided into two groups. The first group consisted of patients treated with short-acting EPO (epoetin-*α*, epoetin-*β*), while the second group consisted of patients who received long-acting EPO (darbepoetin-*α*) for the treatment of anemia. The criteria for application of short- or long-acting EPO was evaluated by physician's assessment in accordance with current recommendations prescribed by the KDIGO Clinical Practice Guideline for Anemia in Chronic Kidney Disease and KDOQI US Commentary on the 2012 KDIGO Clinical Practice Guideline for Anemia in CKD [2, 3]. Depending on the EPO resistance index, both groups of patients were further divided into two subgroups. The first subgroup consisted of patients who were not proved to be resistant to EPO (nonresistant), while the second subgroup consisted of patients who were found to be resistant to EPO (resistant), according to ERI values.

### 2.3. EPO Resistance Index (ERI)

Short-acting ERI is calculated using the following formula ERI (IU/kg/gHb) = [weekly weight − adjusted EPO dose (IU)/hemoglobin concentration in blood (g/L)]. Short-acting EPO resistance exists if the ERI is ≥1.0 IU/kg/gHb. The long-acting ERI is calculated using the following formula: ERI (*μ*g/kg/gHb) = [weekly weight − adjusted EPO dose (*μ*g)/hemoglobin concentration in blood (g/L)]. Resistance to long-acting EPO exists if the ERI is ≥0.005 *μ*g/kg/gHb.

### 2.4. Blood Sampling

Blood samples were obtained from the patients in midweek HD session, before starting the procedure and before administration of heparin. After the session completed, the patient body weight was measured, and blood samples were taken from the patients, in order to determine serum urea concentration and creatinine for further estimation of HD adequacy. Serum and plasma were separated from blood samples for further biochemical analysis. Sera were obtained after centrifugation the blood allowed to clot at room temperature for 2 hours. Plasma samples were obtained after centrifugation the blood collected in anticoagulant tubes, while the erythrocyte lysates were prepared by washing the erythrocyte suspension 3 times in ice-cold saline, following lysis in 3 volumes of ice-cold distilled water. Serum, plasma, and erythrocyte lysates samples were stored at -20°C for further biochemical analysis.

### 2.5. Biochemical Analysis

In order to evaluate the effects of OS, microinflammation, nutrition, and secondary hyperparathyroidism on the development of EPO resistance, different biochemical parameters were investigated. OS parameters were determined from the plasma samples: superoxide anion radical (O_2_^−^), hydrogen peroxide (H_2_O_2_), thiobarbituric acid reactive substances (TBARS), nitrites (NO_2_^−^), and from erythrocyte lysates: superoxide dismutase (SOD) activity, catalase (CAT) activity, and reduced glutathione levels (GSH). The routine biochemical laboratory analysis were performed for determination: hemoglobin (Hb), hematocrit (Hct), iron, iron transferrin saturation (TSAT), ferritin (FER), C-reactive protein (CRP), albumin (ALB), prealbumin (PALB), transferrin (TRSF), intact parathormone (iPTH), and vitamin D levels in sera.

#### 2.5.1. Evaluation of OS Parameters

Determination of OS parameters was previously described [8]. Briefly, the basic principle for determination the concentration of O_2_^−^ in blood plasma samples was based on the reaction of nitro blue tetrazolium in TRIS buffer. Concentration of O_2_^−^ was measured spectrophotometrically at 530 nm. Determination of H_2_O_2_ concentration was based on the oxidation of phenol red by the hydrogen peroxide in reaction catalyzed by horse radish peroxidase. The level of H_2_O_2_ was measured at 610 nm. The degree of lipid peroxidation in the plasma samples was estimated indirectly by measuring TBARS. The principle of this method was determination of lipid peroxide levels in reaction of malondialdehyde (MDA) with thiobarbituric acid, and spectrophotometrically determination was performed at 530 nm. Decomposition of NO and forming stable metabolite nitrite/nitrate compounds were used in the Griess reaction for detection nitrate and NO_2_^–^ levels. The principle of this method involves the use of a Griess reagent, which builds a diazo complex with nitrites giving the purple color. NO_2_^–^ was measured at 550 nm of wave length. An adrenaline method was used to determine the activity of SOD. The principle of this method is to monitor the reduction in the self-oxidation rate of adrenaline in the alkaline environment, which is dependent on O_2_^-.^. Considering that O_2_^−^ was removed by SOD, the adrenaline autoxidation reaction is inhibited. The system monitors the rate of adrenaline autoxidation change through the change in absorbance at 480 nm, which is inversely proportional to SOD activity. The Beutler method was used to determine the CAT activity. The principle was the spectrophotometric monitoring of the rate of decomposition of hydrogen peroxide in the presence of catalase at a wavelength of 230 nm, in which hydrogen peroxide absorbs light. The level of reduced glutathione (GSH) was determined based on GSH oxidation via 5,5-dithiobis-6,2-nitrobenzoic acid. GSH extract was obtained by combining 0.1 mL 0.1% EDTA, 400 *μ*l hemolysate, and 750 *μ*l precipitation solution (containing 1.67 g metaphosphoric acid, 0.2 g EDTA, and 30 g NaCl and filled with distilled water until 100 mL; the solution is stable for 3 weeks at +4 °C). After mixing in the vortex machine and extraction on cold ice (15 min), it was centrifuged at 4000 rpm (10 min). Distilled water was used as a blank probe. The level of GSH was measured at 420 nm. The concentration is expressed as nanomoles per milliliter of red blood cells.

#### 2.5.2. Determination of Serum FER and CRP Concentration

The serum FER concentration was determined by the turbidimetric method, using the Beckman Coulter AU680 apparatus. In patients treated with regular HD, the normal serum FER concentration was considered 100-500 ng/mL. The serum CRP concentration was determined by the turbidimetric method on the Olympus AU680. Normal serum CRP concentration was ≤5 mg/L.

#### 2.5.3. Evaluation of Secondary Hyperparathyroidism Parameters

The concentration of vitamin D in the serum was determined by electrochemiluminescence, using Cobas e411 analyzer. The normal vitamin D concentration in the serum is 20-40 ng/mL. In patients treated with regular HD, the normal vitamin D concentration is ≥30 ng/mL (30-80 ng/mL). A severe deficit exists if the concentration of vitamin D <10 ng/mL, vitamin D deficiency is present if the concentration is 10-20 ng/mL, and the insufficiency is defined as the concentration of vitamin D in the serum of 20-30 ng/mL.The concentration of intact parathormone (iPTH) in the serum was determined by the immunodiathymetric method, on the gamma counter WALLAC WIZARD 1470. The normal levels of iPTH in the serum are 11.8-64.5 pg/mL, while in HD patients, the upper normal concentration is estimated as 300 pg/mL.

#### 2.5.4. Evaluation of Nutritional Status

Folate and vitamin B_12_ concentrations were determined using the Access 2 analyzer by Beckman Coulter via chemiluminescent immunoassay. Prealbumin and transferrin were determined on the Abbott Architect analyzer using the immunoturbidimetric method. Normal concentration of prealbumin in HD patients is ≥0.30 g/L (≥30 mg/dL). The normalized protein catabolic rate (nPCR) is calculated with the following formula, *nPCR* = (*PCR* × 0.58)/*Vd*, where *PCR* is protein catabolic rate and *Vd* is the volume of fluid in the body. PCR is calculated with the following formula, *PCR* = [(9.35 × *G*) + (0.29 × *Vd*)], where *G* is the degree of urea formation, while *Vd* is the volume of fluid in the organism. The degree of urea formation is calculated with the following formula, *G* = [(*C*1 − *C*2)/*Id*] × *Vd*, where *C1* is the concentration of urea in the serum before HD (mmol/L), *C2* is the concentration of urea in the serum after HD (mmol/L), and *Id* is the time between two HD (h). Volume of fluid in the organism is calculated with the following formula, *Vd* = 0.58 × *DW*, in which *DW* represents patient's body weight after HD (kg). The percentage of interdialytic weight gain of patients (%IDWG) is calculated with the following formula: %IDWG = [(body weight of the patient before HD (kg) − patient′s body weight (kg)/patient′s body weight (kg)] × 100].

### 2.6. Assessment of HD Parameters

The HD adequacy was assessed on the basis of the single-pool Kt/V_sp_ index calculated according to Daugridas second-generation formula,*Kt*/*V*_sp_ = −ln (*C*2/*C*1 − 0.008 × *T*) + (4 − 3.5 × C2/C1) × *UF*/*W* (mmol/L), where *T* is the HD duration (h), *UF* is the interdialysis yield (L), and *W* is the body weight after the HD (kg). According to K/DOQI guidelines, *HD* is adequate if Kt/V_sp_ ≥1.2.

The urea reduction rate (URR) index is calculated using the following formula, *URR* = (1 − *R*) × 100%, where *R* represents the ratio of urea concentration in the serum after and before the HD treatment. *HD* is adequate if the URR index =65-70%.

Blood flow through the vascular approach (*Q*_avf_) was determined by the Color Doppler ultrasound scan, on the Logic P5 apparatus, using a 7.5 MHz probe, wherein the blood flow is calculated from the following formula, *Q*_avf_ = *r*^2^*π*/4 × *V*_mean_ × 60 (mL/min), where *r* is the radius of vascular access and *V*_mean_ is the mean blood flow velocity through vascular approach. The blood flow is calculated as the mean of three measurements, 2-4 cm on the vein vascular approach, proximal to the anastomosis site. The blood flow through a vascular approach that provides adequate HD is 500-1000 mL/min.

### 2.7. Statistical Analysis

The data were checked for normality using the Kolmogorov-Smirnov test. Differences between groups were examined using Student's *t*-test and Mann–Whitney *U* test. Probability values of 0.05 and 0.01 were considered statistically significant.

## 3. Results

### 3.1. Oxidative Stress Parameters in EPO Nonresistant and Resistant Patients

The mean values of the studied OS parameters are shown in Tables 1 and 2. The patients with resistance to short-acting EPOs have significantly (*p* < 0.01) lower erythrocyte CAT activity compared to patients without EPO resistance as shown in Table 1.

Patients with resistance to long-acting EPO had significantly (*p* < 0.05) higher serum O_2_^−^ and H_2_O_2_ concentration than those without resistance as shown in Table 2.

### 3.2. Anemia, Iron Status, Microinflammation, Nutritional Status, and Secondary Hyperparathyroidism in Short- and Long-Acting EPO Treatment

The mean values of the parameters of anemia, iron status, microinflammation, nutritional status, secondary hyperparathyroidism, and hypervolemia, depending on the use of short-acting and long-acting EPO, are shown in Tables 3 and 4. Statistically significant lower serum concentration of Hb and Hct levels, as well as higher FER concentration, were registered in patients resistant to short-acting EPO (*p* < 0.01, *p* < 0.01, and *p* < 0.05, respectively), as shown in Table 3. On the other hand, results represented in Table 4 showed that the patients resistant to long-acting EPO had similar FER levels, while Hb values and Hct were significantly lower than nonresistant controls. Also, the mean corpuscular Hb concentration was reduced in group resistant to long-acting EPO. In this group of patients, CRP levels were increased (*p* < 0.05), while PALB and vitamin D concentration were decreased (*p* < 0.01).

### 3.3. General Data on Patients

A total number of 96 patients (58 males, 38 females), mean age of 62.57 ± 11.05 years, mean dialysis treatment length of 4.46 ± 5.22 years, mean body mass index of 25.84 ± 4.93 kg/m^2^, and mean HD adequacy index spKt/V of 1.20 ± 0.28 were examined. General data on patients are presented in Table 5.

From the total 96 patients, 51 were treated with short-acting EPOs, and 45 were treated with long-acting EPO. 12 patients (23.53% of 51) were found to be resistant to short-acting EPOs, and 24 patients (53.33% of 45) were found to be resistant to long-acting EPO. Sensitivity to EPO was found in 39 patients (74.41% of 51) treated with the short-acting EPOs and 21 patient (46.67% of 45) treated with the long-acting EPO.

Regarding sex, 24 male and 15 female patients were nonresistant in short-acting EPO group, while 7 male and 5 female patients were resistant to EPO. In long-acting EPO group, 14 male and 7 female patients were nonresistant to EPO treatment, while 13 male and 11 female patients were EPO resistant.

Short-acting and long-acting EPOs, *i.v.* iron preparations, *i.v.* vitamin B preparations, and folic acid (*per os*) were used for the treatment of anemia of the patients studied. The average monthly dose of short-acting EPO was 20784.31 ± 10673.92 IU and for long-acting EPO 134.89 ± 71.88 *μ*g. The average monthly dose of *i.v.* administered iron, vitamin C, and vitamin B_12_ were 283.02 ± 174.02 mg, 1406.25 ± 196.18 mg, and 3125.00 ± 1986 *μ*g, respectively. The average monthly number of ampoules of vitamin B preparations was 11.25 ± 1.57, and the average monthly dose of folic acid was 190.63 ± 67.01 mg. The secondary hyperparathyroidism of the patients studied was treated with calcium-containing phosphate binders, active vitamin D metabolites, and paricalcitol. The average monthly dose of rocaltrol was 5.33 ± 3.27 *μ*g, and *i.v.* paricalcitol 35.00 ± 20.82 *μ*g.

From the total of 96, 85 patients were treated with standard intermittent high-flux HD, while 11 patients were treated with post-dilution online hemodiafiltration. There were 44 and 41 of patients on HD treatment regarding short- and long-acting EPO administration, respectively. There were 7 and 4 of patients on hemodiafiltration treatment, regarding short- and long-acting EPO administration, respectively.

In 87 patients, HD solution PGS12 was used, in 6 patients PGS25, while only in 3 patients PGS27 was used. Sodium concentration in HD solution was 140 mmol/L, bicarbonate concentration was 35 mmol/L, and K^+^ concentration was 2.0 mmol/L.

The primary kidney diseases, which led to ESRD, are listed in Table 5. The most frequent comorbidity, in both short- and long-acting EPO-treated groups, was arterial hypertension.

Minimal duration on HD treatment was 0.33, 0.33, 0.33, and 0.42 for the nonresistant short-acting EPO, resistant short-acting EPO, nonresistant long-acting EPO, and resistant long-acting EPO group, respectively.

## 4. Discussion

Disturbed oxidative balance, microinflammation, malnutrition, vitamin D deficiency, and increased serum FER concentrations are significant risk factors for the development of EPO resistance in patients undergoing regular HD. In our study, resistance to short-acting EPO was present in 23.53% of patients treated by regular HD. Patients with resistance to the short-acting EPO had a significantly higher serum FER concentration and a significantly lower CAT activity in erythrocytes. Resistance to long-acting EPO was present in 53.33% patients. Patients with resistance to long-acting EPO had a significantly lower concentration of serum PALB and vitamin D, as well as a significantly higher concentration of serum CRP, superoxide anion radical, and hydrogen peroxide compared to patients without resistance.

Predictors of poor outcome in ESRD patients undergoing HD represent OS and microinflammation. Moreover, OS and microinflammation play a major role in the development of EPO resistance. OS occurs due to increased generation of oxygen free radicals and/or decreased activity of antioxidant protection systems. In the HD patients, different pathophysiological mechanisms considering OS play pivotal role in general outcome of ESRD. Furthermore, indicative consideration relies on the claim that vitamin E-coated dialysis membrane improves ERI [9]. The results of our study indicate that patients with resistance to long-acting EPO had significantly higher concentrations of O_2_^−^ and H_2_O_2_, while reduced antioxidant CAT activity was registered in patients with resistance to short-acting EPO. Regardless from EPO resistance and beside the plethora of negative impacts, it was previously reported that oxidative damage in HD patients could impair genomic structure, through DNA-fragmentation induced by increased production of free radicals [10]. This is in accordance with the results of other authors, who found significant positive correlation between hemoglobin concentration in blood and SOD activity in erythrocytes, as well as a significant negative correlation between Hb concentration in blood and MDA concentration in erythrocytes [2]. Moreover, same study revealed lower CAT activity in poor rhEPO responders, which strongly correspond to our results reflecting lower CAT activity in patients with resistance to short-acting EPO (Table 1). Lower activity of antioxidant enzymes, such as CAT, could be discussed as depleted capacity of protective mechanisms in combat oxidative damage and maintaining cellular redox homeostasis. However, the results from literature concerning the effects of HD on OS were contradictory. Study concerning pre- and post-HD levels of antioxidant capacity showed increased CAT activity in diabetic HD patient, as well as SOD [11], suggesting that HD improves antioxidant capacity. However, other authors revealed no difference in levels of antioxidant enzymes [12], and different results could be attributed to characteristics of dialysis membrane. Nevertheless, our focus was not the evaluation of dialysis membrane effects on OS parameters, but rather distinguishing presence of EPO resistance in HD patients regarding OS markers. In our study, resistance to long-acting EPO did not reveal any alteration in estimated antioxidant enzymes activities (SOD and CAT), as well as reduced glutathione levels (Table 2). On the other hand, levels of O_2_^−^ and H_2_O_2_ were significantly elevated in these patients, contrary to short-acting EPO-treated patients. These two prooxidants act as precursors for more reactive species, such as peroxinitrite, hydroxyl radical, or hypocaloric acid, which could disturb cell redox signaling and affect homeostasis in poor long-acting EPO responders. On the other hand, in short-acting EPO-resistant patients, there were no fluctuations in ROS between nonresistant and resistant patients, but lower CAT activity in poor responders have been present, as mentioned above. However, taken together, in both, short- and long-acting resistance to EPO, there was estimated particular oxidative disbalance, which could be responsible to EPO-resistant anemia in HD patients.

OS and microinflammation represent the significant risk factors for the development of EPO resistance in HD patients. The proposed mechanisms suggest that OS and proinflammatory mediators block the proliferation and differentiation of an erythrocyte precursor cells, reduce secretion of endogenous EPO, shorten erythrocyte lifetime, stimulate hepcidin synthesis and secretion in hepatocytes, and cause functional iron deficiency [13]. Optimal control of OS and microinflammation involves the use of biocompatible high-flux dialysis membranes, vitamin E-coated HD membranes, as well as ultra-pure HD solution and online post-dilution hemodiafiltration [7, 14–18]. These data suggest that the usage of zinc, selenium, and vitamin E supplements as strong antioxidant factors modifies OS parameters in HD patients. Our protocol did not include such supplementation, which at first line eliminate influence of antioxidant treatment on estimated OS parameters. However, our results, suggesting the prooxidative state in HD patients regarding decreased SOD or increased O_2_^−^ and H_2_O_2_ (Tables 1 and 2), put in favor antioxidant treatment in regard to prevent oxidative damage. However, these contentions have not been in focus of our investigation, and further studies are needed for understanding the role such antioxidative protocols in HD patients.

It was shown that EPO resistance represents the valuable contributor for increased risk cardiovascular morbidity and mortality [19]. Intriguing data regarding the differences of long versus short-acting EPO treatment in HD patients was recently reported in the Japanese study [20]. These data opposed the US-based cohort [21] results suggesting that long-acting EPO administration could be related to higher mortality rate in HD patients. However, these claims were abolished by Karaboyas et al. who found similar mortality rate as patients prescribed short- versus long-acting EPO [22]. These conflicting results on the use of short versus long-acting EPO strongly suggest that more researches like ours are needed to find specific differences between EPO resistance status of patients with different applied EPOs and some specific biochemical markers such as OS parameters in that patients. In line with that, to our knowledge, our study showed for the first time these specific alterations of OS parameters in EPO recipients regarding their resistance status.

The primary cause of anemia in chronic kidney disease patients represents a decrease in EPO synthesis [23, 24]. The target hemoglobin levels (110-120 g/L) were not achieved in 5-10% of HD patients, despite the appropriate dosage of EPO [25–27]. In line with aforementioned fact, in our study, the average Hb values decreased regarding presence of EPO resistance, in both kinds of treatment (8.69% and 10.36% for short- and long-acting EPOs, respectively), as shown in Tables 4 and 3. Although it seems to be not clinically relevant, those differences maintained Hg levels below 100 g/L in EPO-resistant groups. Anemia could activate compensatory hemodynamic mechanisms to increase blood flow and left ventricle overload, contributing to pathophysiological mechanisms behind functional and structural remodeling of heart and vessels in HD patients [28]. However, the results of our study indicate the higher percentage of EPO resistance in long-acting EPO-treated patients. The result reported by Khalil and coworkers identified a significant negative correlation between Hb levels and inflammatory parameters such as CRP, MDA, and IL-6 [2]. Moreover, other studies showed similar results, with proven significant negative correlation between CRP values and proinflammatory cytokines (such as interleukin-6) with Hb concentration in the blood [29]. Beside the role as a part of mosaic in acute inflammation phase, CRP was considered a long-term predictor of cardiovascular risk and mortality, and its relation with other dialysis-associated concerns was investigated frequently in HD patients. Patients resistant to long-acting EPO from our study had a significantly lower Hb concentration in the blood, as well as significantly higher serum CRP concentration. However, our study did not reveal higher CRP values in short-acting EPO-resistant patients, while Hb levels were decreased.

Hyporesponsiveness to EPO occurs independently of iron intravenous iron therapy, which could imply that other mechanisms could contribute to resistance [30]. Our results showed that patients with resistance to short-acting EPO (epoetin-*α* and epoetin-*β*) have significantly higher serum ferritin concentration than patients without EPO resistance. These results are in line with those of other authors, who found a significant positive correlation between the EPO resistance index and serum ferritin concentration [31].

Synergistically with EPO, vitamin D contributes to erythropoiesis. However, it was known that almost 75% of ESRD patients experience vitamin D deficiency. All treated group of HD patients in our study showed lowered vitamin D levels. Statistically lowered values were found in resistant patients treated by long-acting EPO compared to nonresistant, while the group of short-acting treated patients showed no difference regarding resistance to EPO. These data highlight clinical relevance of vitamin D supplementation in HD patients.

The protein-energy wasting occurs because of malnutrition (in 30-60% of patients) and increased protein metabolism. Risk factors that promote protein catabolism in patients undergoing regular HD include the following: metabolic acidosis, microinflammation, and OS [32]. The results of our study indicated that patients resistant to long-acting EPO had significantly lower serum prealbumin concentration than patients without resistance. This is in accordance with others who found that EPO-resistant patients had higher concentrations of high-sensitive (hs)CRP and interleukin-6 (IL-6), as well as lowered serum albumin and prealbumin concentrations. Moreover, these authors demonstrated that hsCRP and IL-6 are strong and independent positive predictors of EPO resistance, while albumin and prealbumin concentration had shown to be strong and independent negative predictors of the development of EPO resistance [33]. However, our study did not investigate hsCRP which could be considered a limitation of our research.

Serum albumin level and protein catabolic rate were not significantly differ between resistant and nonresistant subjects in both short- and long-acting EPO-treated groups. These findings could be compared with results of Lopez-Gomez and coworkers [34], who found the relation between low values of ERI and albumin levels, but no relation with the normalized protein catabolic rate. Although albumin has been usually used as reflection of nutritional status, it also reflects inflammation state as a protein of acute inflammation phase. While albumin values were similar in all groups, as well as nPCR, we only could discuss CR*P* values in relation to inflammation. Moreover, a decrease of albumin oxidation by vitamin D supplementation in HD patients could potentiate antioxidant defense and decrease chronic inflammation [13], which all together improve general outcome. Beside the mentioned above, recent studies also highlighted a significant positive association between EPO resistance index with serum ferritin and C-reactive protein, percentage interdialytic weight gain (%IDWG), and continuous usage of angiotensin receptor blockers. Also, it was found that there is a significant negative correlation between EPO resistance index and serum albumin concentration [35].

The previous studies had shown that selenium, zinc, or L-carnitine supplementation after each HD session for 2-3 months increase the capacity of enzymatic antioxidant protection and the status of malnutrition and decrease the resistance to EPO [36–38] which accentuate the fact that OS development leads to EPO resistance, as we concluded regarding the results of our study. Moreover, high-flux HD and post-dilution online hemodiafiltration with vitamin E-coated dialysis membranes significantly reduce OS and microinflammation, increase iron availability for erythropoiesis, reduce EPO resistance, and improve nutritional status, quality of life, and the outcome of patients treated with regular dialysis [39–41], which all together imply role of OS in the development of EPO resistance, as we concluded in our study.

## 5. Conclusion

The main results of this study implied that the decreased erythrocyte CAT activity and increased serum ferritin concentration could be significant risk factors for the development of short-acting erythrocyte resistance in HD patients. Moreover, OS, microinflammation, malnutrition, and vitamin D deficiency represent significant risk factors for developing resistance to long-acting EPO. Early identification of patients with increased risk of developing EPO resistance, promptly administration of appropriate therapy, and individualization of dialysis prescription may provide the opportunity for optimal control of OS, microinflammation, malnutrition, vitamin D deficiency, and iron status. All together, these steps could result in better treatment of anemia and lower the rate of cardiovascular morbidity and mortality and improve the quality of life for patients treated with regular HD.

## Figures and Tables

**Table 1:** Parameters of oxidative stress and resistance to short-acting erythropoietin: epoetin-*α*/epoetin-*β*.

Measured parameters	ERI (IU/kg/week/gHb)		*p* Value
	<1.0 IU/kg/gHb	≥1.0 IU/kg/gHb	
	Nonresistant	Resistant	
	Mean ± SD	Mean ± SD	
O_2_^-.^ (nmol/mL)	1.98 (1.98)	2.64 (6.92)	*p* = 0.095
H_2_O_2_ (nmol/mL)	4.37 ± 1.47	4.66 ± 1.54	*p* = 0.552
TBARS (*μ*mol/mL)	1.09 (0.11)	1.12 (0.10)	*p* = 0.197
NO_2_^−^ (nmol/mL)	4.01 ± 1.38	4.00 ± 1.24	*p* = 0.955
GSH (nmol/mL RBC ×103)	117858.57 ± 18403.35	118499.65 ± 14876.13	*p* = 0.913
CAT (U/gHb ×103)	1.75 (1.25)	1.25 (1.19)^∗^	*p* = 0.030
SOD (U/gHb ×103)	39.03 ± 20.64	28.49 ± 16.83	*p* = 0.114

Values are shown as mean (SD), except for O_2_^−^, TBARS, and CAT, where data were not normally distributed and shown as median (interquartile range). ∗*p* < 0.05. ERI, erythropoietin resistance index; O_2_^-.^, superoxide anion radical; H_2_O_2_, hydrogen peroxide; TBARS, thiobarbituric acid reactive substances; NO_2_^−^, nitrites; GSH, reduced glutathione; RBC, red blood cells; CAT, catalase; SOD, superoxide dismutase, Hb, hemoglobin.

**Table 2:** Parameters of oxidative stress and resistance to long-acting erythropoietin: darbepoetin-*α*.

Measured parameters	ERI (IU/kg/week/gHb)		*p* Value
	<0.005 *μ*g/kg/week/gHb	≥0.005 *μ*g/kg/week/gHb	
	Nonresistant	Resistant	
	Mean ± SD	Mean ± SD	
O_2_^-.^ (nmol/mL)	1.32 (1.81)	2.47 (5.68)^∗^	*p* = 0.043
H_2_O_2_ (nmol/mL)	4.16 ± 1.45	5.36 ± 1.99^∗^	*p* = 0.027
TBARS (*μ*mol/mL)	1.09 (0.11)	1.08 (0.08)	*p* = 0.530
NO_2_^−^ (nmol/mL)	3.88 ± 1.31	3.66 ± 1.32	*p* = 0.574
GSH (nmol/mL RBC ×103)	120471.53 ± 16752.57	121478.41 ± 21132.15	*p* = 0.114
CAT (U/gHb ×103)	2.12 ± 1.26	2.14 ± 1.44	*p* = 0.862
SOD (U/gHb ×103)	32.56 ± 20.59	23.74 ± 16.09	*p* = 0.121

Values are shown as mean (SD), except for O_2_^−^ and TBARS, where data were not normally distributed and shown as median (interquartile range).∗*p* < 0.05. ERI, erythropoietin resistance index; O_2_^-.^, superoxide anion radical; H_2_O_2_, hydrogen peroxide; TBARS, thiobarbituric acid reactive substances; NO_2_^−^, nitrites; GSH, reduced glutathione; RBC, red blood cells; CAT, catalase; SOD, superoxide dismutase, Hb, hemoglobin.

**Table 3:** Biochemical profiles of patients according to short-acting EPO (epoetin-*α*/epoetin-*β*) resistance.

Measured parameters	ERI (IU/kg/week/gHb)		*p* Value
	<1.0 IU/kg/gHb nonresistant	≥1.0 IU/kg/gHb resistant	
	Mean ± SD	Mean ± SD	
Hb (g/L)	104.73 ± 10.07	95.63 ± 9.94	*p* = 0.008
Hct (%)	31.85 ± 3.02	29.10 ± 6.55	*p* = 0.009
MCV (fL)	94.77 ± 4.11	94.34 ± 6.55	*p* = 0.785
MCH (pg)	31.28 ± 1.57	31.10 ± 2.08	*p* = 0.737
MCHC (g/L)	329.94 ± 4.76	329.79 ± 5.89	*p* = 0.931
FOL (ng/mL)	25.80 (12.10)	25.80 (0.00)	*p* = 0.434
VitB_12_ (pg/mL)	1197.00 (1012)	1342.50 (1106)	*p* = 0.925
Fe (*μ*mol/L)	10.43 ± 4.59	10.89 ± 4.43	*p* = 0.762
TSAT (%)	30.05 ± 13.30	32.13 ± 12.00	*p* = 0.632
FER (ng/mL)	723.82 ± 332.64	1016.38 ± 371.10	*p* = 0.015
CRP (mg/L)	4.45 (11.75)	7.32 (19.95)	*p* = 0.297
TP (g/L)	63.83 ± 5.23	63.29 ± 4.20	*p* = 0.745
ALB (g/L)	37.46 ± 2.76	38.25 ± 3.23	*p* = 0.410
PALB (g/L)	0.26 ± 0.09	0.30 ± 0.06	*p* = 0.174
TRSF (g/L)	1.55 ± 0.30	1.49 ± 0.27	*p* = 0.563
UA (*μ*mol/L)	359.24 ± 58.07	336.21 ± 65.54	*p* = 0.249
BMI (kg/m^2^)	25.90 ± 4.89	25.03 ± 3.32	*p* = 0.570
nPCR (g/kg/24 h)	1.69 ± 0.62	1.96 ± 0.49	*p* = 0.181
IDWG (%)	3.07 ± 1.38	3.62 ± 0.95	*p* = 0.209
VitD (ng/mL)	14.83 ± 7.67	18.69 ± 12.62	*p* = 0.200
iPTH (pg/mL)	80.20 (164.40)	115.60 (180.38)	*p* = 0.657
Kt/V	1.03 ± 0.24	1.05 ± 0.13	*p* = 0.780
spKt/V	1.21 ± 0.28	1.25 ± 0.16	*p* = 0.666
URR (%)	63.21 ± 8.72	64.65 ± 5.24	*p* = 0.592
MMDI (mg)	280.00 ± 197.91	228.57 ± 111.27	*p* = 0.518

Values are shown as mean (SD), except for FOL, VitB_12_, CRP, and iPTH where data were not normally distributed and shown as median (interquartile range). ERI, erythropoietin resistance index; Hb, hemoglobin; Hct, hematocrit, MCV, mean corpuscular volume; MCH, mean corpuscular hemoglobin; MCHC, mean corpuscular hemoglobin concentration; FOL, serum folate concentration; VitB_12_, serum vitamin B_12_ concentration; Fe, serum iron concentration; TSAT, transferrin saturation; FER, serum ferritin concentration; CRP, serum C-reactive protein concentration; TP, serum total protein concentration, ALB, serum albumin concentration; PALB, serum prealbumin concentration; TRSF, serum transferrin concentration; UA, serum uric acid concentration; BMI, body mass index; nPCR, normalized protein catabolic rate; IDWG, interdialytic weight gain; VitD, serum vitamin D concentration; iPTH, serum intact parathormone concentration; Kt/V, hemodialysis adequacy index; spKt/V, single-pool index of hemodialysis adequacy; URR, urea reduction ratio; MMDI, mean monthly dose of *i.v.* iron.

**Table 4:** Biochemical profiles of patients according to long-acting EPO (darbepoetin-*α*) resistance.

Measured parameters	ERI (*μ*g/kg/week/gHb)		*p* Value
	<0.005 *μ*g/kg/gHb nonresistant	≥0.005 *μ*g/kg/gHb resistant	
	Mean ± SD	Mean ± SD	
Hb (g/L)	108.64 ± 8.53	97.35 ± 13.42	*p* = 0.002
Hct (%)	32.67 ± 2.64	30.50 ± 4.55	*p* = 0.007
MCV (fL)	93.48 ± 3.79	92.96 ± 4.11	*p* = 0.660
MCH (pg)	30.99 ± 1.30	30.49 ± 1.44	*p* = 0.234
MCHC (g/L)	331.41 ± 4.21	328.60 ± 4.86	*p* = 0.046
FOL (ng/mL)	25.80 (5.80)	25.80 ± 12.60	*p* = 0.570
VitB_12_ (pg/mL)	1500.00 (908)	1304.00 (1060)	*p* = 0.521
Fe (*μ*mol/L)	9.77 ± 3.99	8.49 ± 3.02	*p* = 0.230
TSAT (%)	26.98 ± 9.94	25.48 ± 10.95	*p* = 0.635
FER (ng/mL)	692.60 ± 298.31	767.67 ± 296.22	*p* = 0.403
CRP (mg/L)	7.38 ± 7.47	17.38 ± 18.32	*p* = 0.024
TP (g/L)	65.36 ± 4.73	65.15 ± 5.24	*p* = 0.888
ALB (g/L)	38.81 ± 3.19	37.50 ± 3.58	*p* = 0.205
PALB (g/L)	0.32 ± 0.07	0.25 ± 0.09	*p* = 0.009
TRSF (g/L)	1.67 ± 0.40	1.49 ± 0.37	*p* = 0.126
UA (*μ*mol/L)	372.71 ± 47.51	370.46 ± 50.18	*p* = 0.878
BMI (kg/m^2^)	26.11 ± 5.26	25.93 ± 5.58	*p* = 0.912
nPCR (g/kg/24 h)	1.63 ± 0.46	1.91 ± 0.58	*p* = 0.085
IDWG (%)	3.28 ± 1.76	3.85 ± 1.73	*p* = 0.279
VitD (ng/mL)	24.24 ± 11.26	15.99 ± 8.84	*p* = 0.009
iPTH (pg/mL)	107.08 ± 119.69	179.32 ± 147.55	*p* = 0.081
Kt/V	0.95 ± 0.19	1.02 ± 0.29	*p* = 0.328
spKt/V	1.12 ± 0.22	1.22 ± 0.35	*p* = 0.260
URR (%)	60.50 ± 7.51	62.51 ± 10.33	*p* = 0.466
MMDI (mg)	277.78 ± 130.17	325.00 ± 186.47	*p* = 0.524

Values are shown as mean ± SD, except for FOL and VitB_12_, where data were not normally distributed and shown as median (interquartile range). ERI, erythropoietin resistance index; Hb, hemoglobin; Hct, hematocrit, MCV, mean corpuscular volume; MCH, mean corpuscular hemoglobin; MCHC, mean corpuscular hemoglobin concentration; FOL, serum folate concentration; VitB_12_, serum vitamin B_12_ concentration; Fe, serum iron concentration; TSAT, transferrin saturation; FER, serum ferritin concentration; CRP, serum C-reactive protein concentration; TP, serum total protein concentration, ALB, serum albumin concentration; PALB, serum prealbumin concentration; TRSF, serum transferrin concentration; UA, serum uric acid concentration; BMI, body mass index; nPCR, normalized protein catabolic rate; IDWG, interdialytic weight gain; VitD, serum vitamin D concentration; iPTH, serum intact parathormone concentration; Kt/V, hemodialysis adequacy index; spKt/V, single-pool index of hemodialysis adequacy; URR, urea reduction ratio; MMDI, mean monthly dose of *i.v.* iron.

**Table 5:** General data of the patients, categorized by type of EPO administered and presence of EPO resistance.

General data	Short-acting EPO		Long-acting EPO	
	Nonresistant	Resistant	Nonresistant	Resistant
	Mean ± SD	Mean ± SD	Mean ± SD	Mean ± SD
Age (years)	66.00 ± 9.17	72.17 ± 7.40	60.00 (12.50)	60.00 (11.00)
HD treatment length (years)	2.50 (7.16)	3.67 (4.35)	1.42 (2.54)	3.84 (6.73)
SBP (mmHg)	130.00 (20.00)	130.00 (7.50)	130.00 (20.00)	125.00 (30.00)
DBP (mmHg)	80.00 (10.00)	80.00 (10.00)	80.00 (5.00)	80.00 (10.00)
MBP (mmHg)	96.67 (13.33)	96.67 (9.17)	96.67 (6.67)	93.33 (16.67)
BW (kg)	70.00 (15.00)	66.42 (10.63)	75.40 ± 14.10	69.60 ± 16.96
IDWG (kg)	2.00 (1.50)	2.42 (1.00)	2.48 ± 1.37	2.65 ± 1.21
UR (mL/h)	500.00 (375.00)	625.00 (250.00)	621.03 ± 340.52	661.46 ± 302.79
UR (mL/kg/h)	7.68 ± 3.46	9.04 ± 2.37	8.23 ± 4.36	9.63 ± 4.32
RD (mL/24 h)	500.00 (1000.00)	350.00 (1250.00)	1000.00 (1200)	500 (1000)
Q_avf_ (mL/min)	750.00 (750.00)	800.00 (637.50)	750.00 (990.00)	600.00 (675.00)
PKD				
CG (*N*, %)	3 (7.69)	1 (8.33)	2 (9.52)	2 (8.33)
HN (*N*, %)	13 (33.33)	5 (41.67)	4 (19.05)	6 (25.00)
DN (N, %)	7 (17.95)	3 (25.00)	4 (19.05)	4 (16.67)
ON (*N*, %)	4 (10.26)	0 (0.00)	0 (0.00)	2 (8.33)
CN (*N*, %)	11 (28.21)	3 (25.00)	3 (14.29)	7 (29.17)
PK (*N*, %)	1 (2.56)	0 (0.00)	8 (38.10)	3 (12.50)

Values are expressed as means ± SD for normally distributed data, or as median (interquartile range) where data were not normally distributed. HD, hemodialysis; SBP, systolic blood pressure; DBP, diastolic blood pressure; MBP, mean blood pressure; BW, body weight; IDWG, interdialytic weight gain; UR, ultrafiltration ratio; RD, residual diuresis; Q_avf_, arteriovenous fistula flow; Kt/V, hemodialysis adequacy index; spKt/V, single-pool hemodialysis adequacy index; URR, urea reduction ratio; PKD, primary kidney disease; CG, chronic glomerulonephritis; HN, hypertensive nephropathy; DN, diabetic nephropathy; ON, obstructive nephropathy, CN, chronic nephropathy; PK, polycystic kidney disease.

## Data Availability

The data used to support the findings of this study are available from the corresponding author upon request.
